# A Two-Stage Method to Determine Optimal Product Sampling considering Dynamic Potential Market

**DOI:** 10.1155/2015/167481

**Published:** 2015-03-04

**Authors:** Zhineng Hu, Wei Lu, Bing Han

**Affiliations:** Uncertainty Decision-Making Laboratory, Sichuan University, Chengdu 610064, China

## Abstract

This paper develops an optimization model for the diffusion effects of free samples under dynamic changes in potential market based on the characteristics of independent product and presents a two-stage method to figure out the sampling level. The impact analysis of the key factors on the sampling level shows that the increase of the external coefficient or internal coefficient has a negative influence on the sampling level. And the changing rate of the potential market has no significant influence on the sampling level whereas the repeat purchase has a positive one. Using logistic analysis and regression analysis, the global sensitivity analysis gives a whole analysis of the interaction of all parameters, which provides a two-stage method to estimate the impact of the relevant parameters in the case of inaccuracy of the parameters and to be able to construct a 95% confidence interval for the predicted sampling level. Finally, the paper provides the operational steps to improve the accuracy of the parameter estimation and an innovational way to estimate the sampling level.

## 1. Introduction

For a long time, most researches and applications on the diffusion of a new product have involved the new product growth model proposed by Bass [1] (often referred to as Bass model) and formed the Bass model family or flexible diffusion models by changing the assumptions of Bass model, adding new meanings to the original factors, or introducing the new influence factors [2–6], though there were some other methodologies applied to model the product diffusion process [7–9], such as the agent-based modeling and simulation methodology.

During the product diffusion, free samples together with pricing strategy and repeat purchase behavior influence the diffusion process, and most researches have focused on the situation after market launch. Lammers [10] stated that small sample packages can promote the sales. Moreover, Heiman et al. [11] indicated that the samples increase the probability of consumers' purchasing and promote communication among consumers. As for samples for individual product, Jain et al. [12] studied the optimum proportion of the durable product as samples based on the Bass model. Hu and Li [13] and Hu [14] pointed out that free samples can promote the diffusion of a new product and also analyzed the influence of individual product diffusion caused by free samples under different price strategies. All of these studies were based on one assumption that the potential consumer market is fixed and there is no any change of consumers. However, in reality, the potential consumers keep changing all the time even under the assumption of constant amount of the potential market; and, in each period, new consumers would come into the market, but some potential consumers disappear and do not buy the product anymore.

After Mahajan and Peterson [15] classified four types of diffusion models, in which the number of the potential consumers is set to a constant, Liu et al. [16] stated that the internet product diffusion model for potential consumers is based on a function of time, and the increase of internet users has an impact on the internet products' potential consumers. In product diffusion literature, the change of potential consumers involves four situations: (1) the number of potential consumers is a constant, such as that in Bass model; (2) the number of potential consumers increases with the time; (3) the number of potential consumers decrease with the time; (4) potential consumer is affected by some factors; then potential consumer function can be created in the light of exponential growth correction pointed by Rai et al. [17].

Some researchers pointed out that the function of potential consumer is a time series function, which always related to the product features. Compared to the constant amount of the potential market, this paper presents a new product diffusion model, considering the impact of the external and internal factors; however, there is an assumption that the potential market is dynamic rather than static; namely, in each period, some potential consumers disappear and some new potential consumers come into the market so that the amount of potential market is unchanged in line with previous study assumptions. The paper found out that the dynamic potential market plays a key role in the diffusion of new product. To do this, Section 2 builds a model under the Bass model framework; Section 3 shows a solution approach and then gives a local sensitivity analysis and a global sensitivity analysis, showing that a two-stage statistic analysis would be able to figure out the sampling level directly with high accuracy, which is especially suitable for the sales manager's decision making in a dynamic potential market. Finally, Section 4 makes a concluding remark on the two-stage method for sampling level and presents some possible considerations for future work.

## 2. Model Building

Considering dynamic potential market, this section builds an optimization model on product diffusion with sampling based on Bass model.

### 2.1. Problem Statement

In the presence of product diffusion, some consumers are attracted by advertisement to purchase the product; and the factors, such as oral communication, may make other people who know the product but have not decided whether to purchase it or not become the adopters. Put simply, the model focuses on the nondurable product whose price is not very high, because the cost of sampling is so high for expensive product that no firm would prefer this type of conduction. In this case, sampling can express the features of product to people and make persistence of memory and consumption for the people.

**Figure 1:**
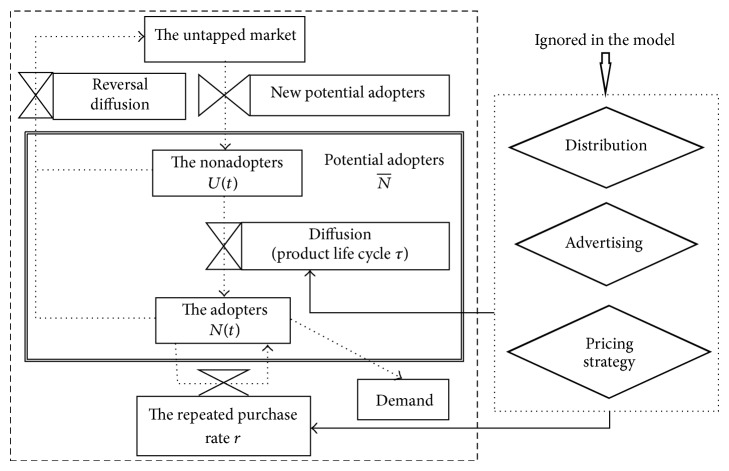
The diffusion process for nondurables.

Generally, the consumers can be divided into two groups. One group consists of the nonadopters, including (1) the people who have ever purchased the product but at the moment have not decided to buy it and (2) the people who never purchase it. Another group consists of the adopters. Both groups are probably transformed into each other, forming a dynamic process of potential market (see Figure 1).

Several assumptions underlie the diffusion model incorporating price, most of which are simplifying assumptions by Mahajan et al. [2] which provide a parsimonious analytical representation of the diffusion process.

1. The geographic boundaries of the social system do not change over the diffusion process.
2. Nature of a product does not change over time.
3. The diffusion process is depicted as the process of a nonadopter being transformed into an adopter.
4. There is no supply constrain.
5. Sample is given only once to one consumer, but consumer can repurchase the prodcut.
6. The diffusion of a new product is independent of other products.

The notations are shown in the Notation section.

### 2.2. Model Development

In recent years, a lot of mathematical models have been proposed to describe the relationship between the new product diffusion process and the S-shaped nature of its adoption curve since the publication of the Bass model [1]. The basic Bass model is shown as a set of discrete dynamical equations:

(1) $$\begin{array}{c} N\left(t+1\right)-N\left(t\right)=\left(a+bN\left(t\right)\right)\left(\bar{N}-N\left(t\right)\right). \end{array}$$

In (1), traditionally, the value for *N*(0) has been assumed to be zero, which is an initial condition.

#### 2.2.1. Impact of Dynamic Potential Market

As potential market is a dynamic process, it assumed that, in each period, new potential consumers enter into the market as a certain percentage of $\bar{N}$, and the loss is the same percentage of *N*(*t*). Although some people become the potential consumer, they have not purchased product before being changed to be nonpotential consumers; therefore, it is dealt as net inflow. The net outflow is equal to a certain percentage of total potential market amount minus the number of new potential consumers. The model describes the first time purchase behavior, so the net inflow and outflow are expressed as follows:

(2) $$\begin{array}{c} n\left(t+1\right)≜N\left(t+1\right)-\left(1-\gamma_{u}\right)N\left(t\right)=\left(a+bN\left(t\right)\right)U\left(t\right),\\ u\left(t+1\right)≜U\left(t+1\right)-\left(1-\gamma_{u}\right)U\left(t\right)=\gamma_{u}\bar{N}-n\left(t+1\right), \end{array}$$

where if 0 < *γ*_*u*_ ≤ 1, actually, $U(t)+N(t)=\bar{N}$ and if *γ*_*u*_ = 0, the model is reduced to the Bass model.

#### 2.2.2. Impact of Product Sampling

Lammers [10] pointed out that free samples can be an effective way to create an initial pool of “adopters,” and this pool along with the regular group of innovators would influence other potential adopters via words of mouth. So the potential market affected by sampling could be expressed below:

(3) $$\begin{array}{c} \gamma_{b}\gamma_{n}\left(t+1\right)n_{f}\left(t+1\right)=N_{f}\left(t+1\right)-N_{f}\left(t\right). \end{array}$$

And (1) changes to

(4) nt+1=N(t+1)−N(t)=a+b(N(t)+Nf(t))(N¯−N(t)−Nf(t)),

where $\gamma_{n}(t+1)=1-(N(t)+N_{f}(t))/\bar{N}$, *γ*_*n*_(0) = 1, and *N*_*f*_(0) = *γ*_*b*_ *n*_*f*_(0). *N*_*f*_(*t*) is the effective sample, which means the people who get free sample minus the nonpotential consumers and adopters. In particular, *γ*_*n*_(*t*) = 1 means accurate sampling.

Inflow and outflow of the potential market with sampling could be expressed below:

(5) $$\begin{array}{c} n\left(t+1\right)=\left[a+b\left(1-\gamma_{u}\right)\left(N\left(t\right)-N_{f}\left(t\right)\right)\right]U\left(t\right),\\ u\left(t+1\right)=\gamma_{u}\bar{N}-n\left(t+1\right)-\gamma_{b}\gamma_{n}n_{f}\left(t+1\right). \end{array}$$

During the diffusion process, the potential market is divided into adopters and free sample recipients. Potential consumers number is the upper limit of the number of the adopters. So the cumulative diffusion volume of product plus the number of potential buyers among those who have accepted samples should be less than the number of potential consumers, which means that

(6) $$\begin{array}{c} N\left(t\right)+N_{f}\left(t\right)\leq \bar{N}. \end{array}$$

#### 2.2.3. Impact of the Repeat Purchase

The product life cycle would make consumers consider repurchase. Meade and Islam [3] drew a conclusion from literature: if every product has a life cycle, especially for nondurable products, repurchase rate of new product *γ* ≤ 1. Usually, a firm hopes that the consumers make a run-ahead consumption of products in order to gain benefits and to reduce uncertainty of future sales volume, so it is necessary to spread diffusion by sampling.

When *t* ≥ *τ*, the number of the adopters in period *t* − *τ* minus the number of repurchasing consumers from *t* + 1 − *τ* to *t* − 1 is the potential repurchasing consumers in period *t*; namely,

(7) $$\begin{array}{c} R\left(t\right)=\left\{\begin{array}{cc} N\left(t-\tau \right)+N_{f}\left(t-\tau \right) & \\ \;-\;r\overset{\tau -1}{\underset{j=1}{\sum }}R\left(t-j\right), & \tau >1,\;\;t>\tau ,\\ N\left(t-\tau \right)+N_{f}\left(t-\tau \right), & \tau =1,\;\;t>\tau ,\\ 0, & t\leq \tau . \end{array}\right. \end{array}$$

The number of sales volume is

(8) $$\begin{array}{c} S\left(t+1\right)=N\left(t+1\right)-N\left(t\right)+\gamma_{b}\gamma_{n}\left(t\right)n_{f}\left(t\right)+rR\left(t+1\right). \end{array}$$

#### 2.2.4. Objective Function

One may also argue that product sampling is expensive for the firm, so it may not be economical to give free samples to every potential adopter. As a result, serious consideration must be given to the question of how many samples would be distributed. In addition, offering too many free samples would cost the firm's resource. The firm needs to determine the “right” amount of sampling. Then, one can get the objective function to maximize the net present value (NPV) of the firm given by

(9) π=∑t=1T11+irt(p(t)−c)S(t)−(h+c)nf(t) −(h+c)nf(0),

where *h* is the cost of unit sampling, including the labor, packaging cost, transportation costs, and handling costs; *c* is unit cost of product.

#### 2.2.5. The Model

Taking all the factors above into consideration, a model can be established to provide an analytical framework for incorporating explicitly the effect of product sampling on product diffusion. That is,

(10) max⁡ πsubject  to (3)~(9),

where the decision variables are *n*_*f*_(*t*).

## 3. Analysis and Discussions

Model (10) is a nonlinear optimization problem. In reality, potential market is a dynamic process, which means that, in each period, some potential consumers would disappear and some new ones would come into being, although, just like Bass and the followers have done, the total number of the potential markets is assumed to be constant. Considering dynamic changes of potential market, the firm should develop marketing strategies in line with diffusion trend, so this section focuses on sampling marketing strategy, firstly discussing the impact of sampling rate on product diffusion and then including the repeat purchase to find out the most favorable sampling rate.

When new product is put on the market, the firm would carry out some marketing strategies, such as the sampling, which is practical, simple to operate, and effective. How to play the role of sampling policy and how the firm determining sampling rate to achieve the maximal profits according to the actual situation would be discussed here. To facilitate comparative analysis, some parameters are defined as follows [12, 18]: *a* = 0.02, *b* = 0.35, $\bar{N}=54$; *c* = 1, *c*_*f*_ = 1, *h* = 1, *r* = 0.5, *τ* = 2, *λ*_*b*_ = 0.1, *λ*_*u*_ = 0.02, *p* = 10, and *i*_*r*_ = 0.008. During the diffusion process, the potential market is assumed to be constant and the firm's goal is to maximize the profit; hence, the analysis of sampling's impact on the diffusion would be made. The optimal solution is first gotten by LINGO, and the sensitivity analysis would be conducted later by MATLAB and SAS.

To analyze the stability of the sampling level when parameters are changed, this section mainly analyzes the relationship between the parameters and the sampling level. The reasons to do this are mainly stated below. Firstly, there exists differences in the sensitivity of the model to the parameter and the requirements of the parameter estimation accuracy. Secondly, systematic effects due to the parameter sensitivity change, debugging of the parameters from the application issues and making the classification design to the decision schemes. This work would allow one to understand, under which conditions of parameter values, the NPV and sampling level could be the best, using statistical analysis to make a comparative analysis of the relevant parameters. Hyman [19] said that the acceptability of parameter estimation procedures is dependent upon the error sensitivity of these models. How various parameters combination affects the sampling level and which parameters have greater influence on the model would be considered below.

### 3.1. Parameters Involved

The external coefficient, *a*, and internal coefficient, *b*, are both parameters that can influence the diffusion rate of the new product. Because of the impact of different advertising levels, the external coefficient and internal coefficient appear different. Sultan and Farley [20] gave a metal-analysis of applications of diffusion models with 213 groups data in 15 papers shown below: for the analyses fitting the equations above, the maximal coefficient of external influence is 0.23 and the minimal one is 0.00002; the maximal coefficient of external influence is 0.99 and the minimal one is 0.00003. Considering the actual situation of product sales, the extreme cases of external coefficient and internal coefficient would not exist, which is the parameter setting for local sensitivity analysis. Therefore, to have a comprehensive analysis of the parameters, the parameters and classification levels are shown in Table 1 for global sensitivity analysis.

In total, there are 5^4^∗3 = 1825 treatments. For each experiment running, MATLAB would output the NPV and sampling level at a time. The average NPV difference is $19.81 million, with a minimum, median, and maximum NPV of $83.05 million, $473.17 million, and $1974 million. Then, the difference between NPV and sampling level for each of the 1875 diffusion processes is calculated. Finally, the statistical analysis is conducted to understand the relationship between the experimental parameters and the NPV and sampling level. In particular, the regression analysis is shown with the experimental parameters as independent variables to analyze the impact of each parameter on NPV and sampling level through the *t*-statistic for its respective regression coefficient. Moreover, removing the nonsampling sample cases, a linear regression analysis of the sampling level determination for sample cases is presented. The results show that the determination between the experimental parameters and dependent variables is greatly increased in this way.

### 3.2. Impact of Key Factors on the Sampling Level

**Figure 2:**
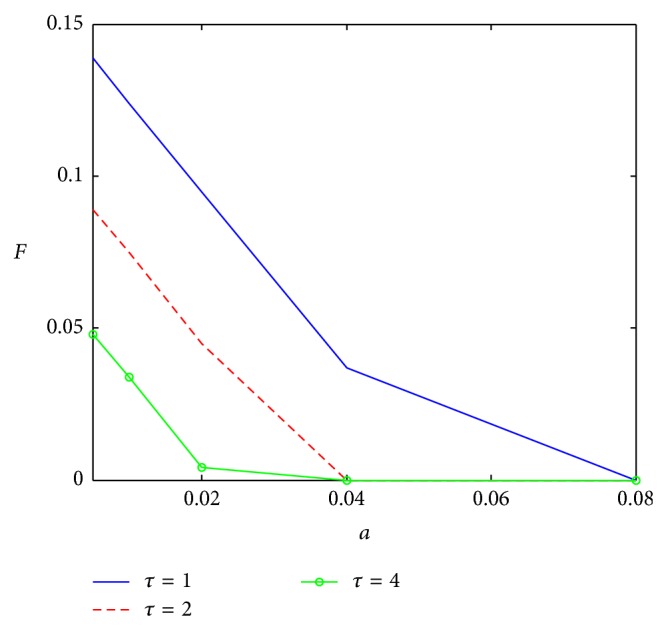
Impact of *a* on sampling level.

**Figure 3:**
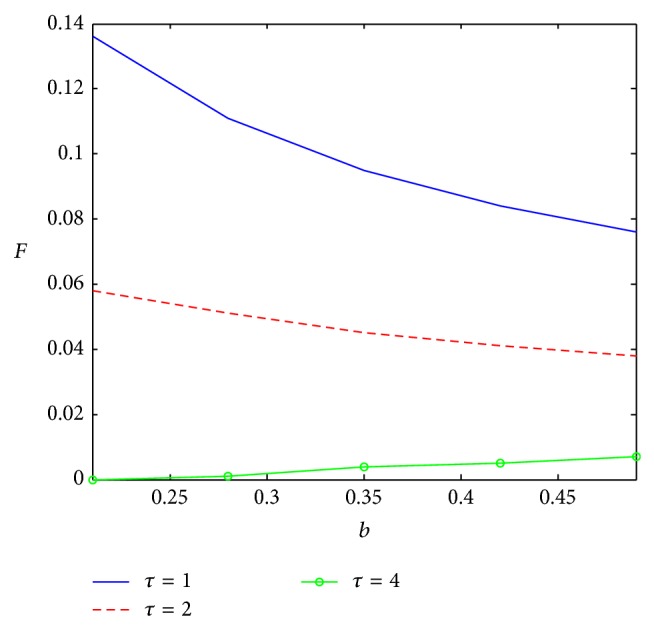
Impact of *b* on sampling level.

Aimed at the impact of the key factors, Figure 2 shows the influence of external coefficient, *a*, on sampling level; with the increase of external coefficient, sampling level is declining. Given higher value for the external coefficient, there would be enough innovators to adopt the product; so an attempt to generate additional innovators through sampling may be a waste of resources. The sampling level would also decline with the internal coefficient increasing (Figure 3). If the internal coefficient is large enough, oral communication would promote the diffusion of product so that more samples only increase the cost. As shown already, three curves, respectively, express the sampling levels with different product life cycle. With shorter life cycle, the firm deserves more sampling to get more adopters, those who would repeat purchase. As repeat purchase occurs more frequently with shorter life cycle, the firm would prefer using more sampling to catch the consumers, considering the cost of sampling. However, with a longer life cycle, if internal coefficient is large enough, more sampling would promote the diffusion.

**Figure 4:**
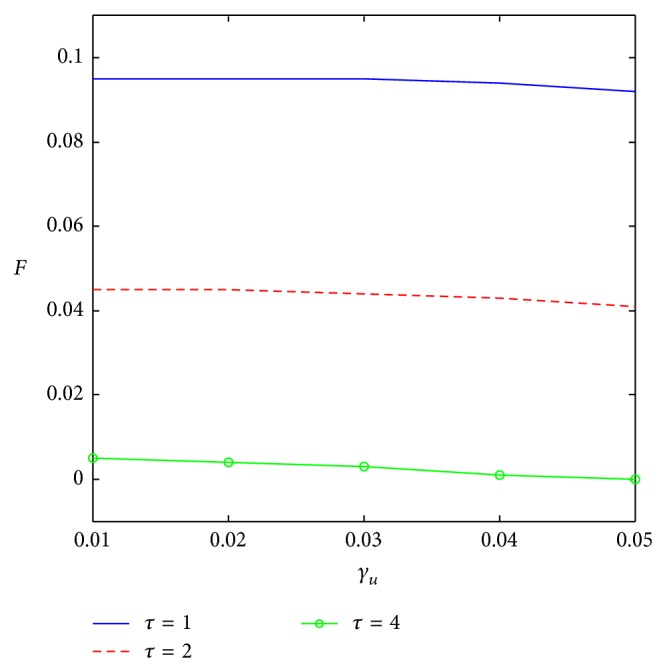
Impact of *γ*_*u*_ on sampling level.

**Figure 5:**
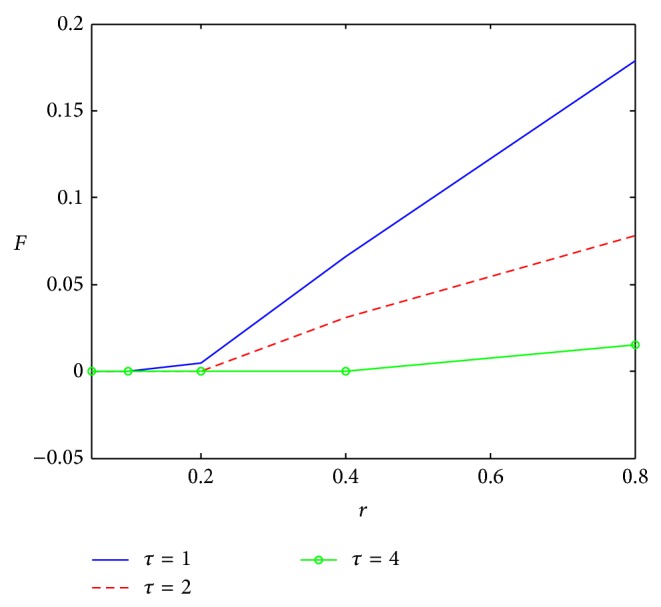
Impact of *r* on sampling level.

Figure 4 shows that the change rate of potential market has little effect on sampling level. With the increase of *γ*_*u*_, sampling level changes a little under three types of life cycles. When product life cycle is beyond a certain value, sampling would not improve the profit. This is because, after first purchase, there needs a long time for repeat purchase and oral communication could have an enough influence on the spread of the product itself. The effect of extra sampling is not obvious and the sampling level would not increase anymore. Repeat purchase rate has great influence on sampling level (Figure 5). The higher the repeat purchase rate is, the higher the sampling level is. Higher repeat purchase means that more consumers would repeat purchase after first purchase. So, in earlier periods, the spread of the product should catch more consumers.

### 3.3. Two-Stage Method Determining the Sampling Level

To present a two-stage method to determine the sampling level, this subsection turns to global sensitivity analysis and runs a full-factorial experimental design with five parameters at five levels each to develop further insights into the impact of the parameters on the whole model.

#### 3.3.1. The Level of the Key Factor's Influence

Testing the correlation between the parameters and NPV and sampling level, Table 2 gives Pearson Correlation Coefficients Test first. some conclusions could be drawn below. (1) External coefficient *a* and internal coefficient *b* have a positive influence on the NPV; however, as for the sampling level, both of them have a negative influence. (2) Variables of *a*, *b*, and *r* are significant at *P* < 0.0001, which shows the strong correlation between the parameters *a*, *b*, and *r* and the product sampling level. (3) For *γ*_*u*_, it is not significant with sampling level because the *P* value is equal to 0.7224. (4) The product life cycle has an uncertain correlation with sampling level of the product because the *P* values of *τ* (one-period life cycle, disposable goods) and *τ*_4_ (four-period life cycle, least disposable goods) are far less than 0.05, whereas the *P* value of *τ*_2_ (two-period life cycle, less disposable goods) approaches 0.05.

Therefore, using the 1875 experimental data, two multiple linear regressions are presented with the sampling level as a dependent variable and the parameters as the independent variables in Table 3 and with NPV as a dependent variable and the parameters and sampling levels as the independent variables in Table 4. The tables present the *t* value and the significance levels to summarize the level and direction of the particular variable's influence. All variables are significant at *P* < 0.0001, which again confirms the above estimated correlations.

1. The adjusted *R*-square of the five parameters to sampling level is 0.5511, indicating that the effect of interaction between them is relatively strong. On the other hand, the adjusted *R*-square of the parameters to NPV is 0.8173, which represents a strong interaction between them.
2. Given by their *t* values, parameters are ordered as *r* > *a* > *τ*_4_ > *τ*_2_ > *b* with respect to their influences on the sampling level differences. With respect to NPV level, the order is *r* > *τ*_4_ > sl > *a* > *τ*_2_ > *b* > *γ*_*u*_. This indicates that, in the diffusion process, repeat purchase rate and product life cycle are the two most influential parameters to influence the NPV and sampling level of the new products.

#### 3.3.2. Logistic Regression for Sampling or Not

However, the regression models above cannot be sufficient to fully explain the sampling level differences because there are large number of experiments without sampling. Actually, among 1875 sets of experimental data, only 727 sets of them show the needs for sampling. To make more accurate analysis, logistic regression analysis is used to distinguish whether to need sampling or not, followed by linear regression discussed later if sampling needed.

Here, sampling or not could be a discrete variable as dependent variable of logistic regression model, defining a dependent variable, sampling level (sl), which means that sampling level is 1 if sampling is needed and is 0 otherwise. To use the logistic regression model for discrimination purposes, compute an estimate of the probability that sl is equal to 1 given the values of the predictor variables to decide whether to need sampling or not. If the estimated probability is less than 0.5, the sampling is needed.

The variables selected in the sensitivity analysis include continuous variables: the diffusion parameters (external coefficient *a* and internal coefficient *b*), change rate of potential market *γ*_*u*_, and the parameters of repeat purchase *r* and discrete variables: product life cycle *τ*.

The chi-square test in Table 5 indicates that the predictor variables being used are statistically significant predictors. The test considers all predictors variables simultaneously.

Table 6 contains the classification summary matrix for 1875 sets of data. The furthest left column shows the group of sampling or not, and the labels on the top show the groups of whether sampling or not. One can see that 949 of 1036 (91.60%) of the data without sampling would be classified correctly by the logistic discriminant rule, and that 762 of the 839 (90.8%) sets of data with sampling would be classified correctly.

Table 7 lists the parameter estimates of the parameters in the logit function. Logistic regression analysis gives a probability to decide whether to need sampling or not. It also expresses how the variables affect the probability, respectively.

#### 3.3.3. Linear Regression for Sampling Level

The 839 sampling experiments are taken out from the total 1875 experiments to do a multiple linear regression.  Table 8 is the statistics of multiple linear regression for selected sampling experiments, showing that all variables in the regression model are significant at *P* < 0.0001.

(1) The adjusted *R*-square increases significantly from 0.5511 to 0.7472 which illustrates that the relevance of the parameters and the sampling level has improved a lot and the adjusted model fits the sampling level very well. Meanwhile, through the statistics analysis of NPV with the 839 selected experiments, the adjusted *R*-square also increases significantly from 0.8173 to 0.9287, which suggests that the selected experiments fit the NPV perfectly as well.
(2) It can be seen from Table 8 that the parameter estimates of *τ*_2_ and *τ*_4_ are −0.04801 and −0.0809 which just shows the gap of sampling levels between *τ*_1_ and *τ*_2_, *τ*_1_, and *τ*_4_ in Figure 4. Due to the irrelevance of *γ*_*u*_ and sampling level, these data are more convincing to show that this model fits the sampling data very well.
(3) The ratio of Predicted Residual SS (0.91487) and Sum of Squared Residuals (0.90009) is 1.0164, approaching 1, which also indicates that the adjusted model is more accurate than the earlier one.
(4) Both estimated values of *τ*_2_ and *τ*_4_ are negative, and the parameter value of *τ*_4_ is larger than that of *τ*_2_. So, with the value of *τ* increasing, sampling level will decline, which is the same as local sensitivity analysis.
(5) The regression model for sampling level being as a dependent variable is below:
  (11) sl=0.09514−2.5145a−0.12693b+0.18473r −0.04801τ2−0.0809τ4.

After substituting the original parameters (*a* = 0.02, *b* = 0.35, *r* = 0.5, *τ*_2_ = 1, and *τ*_4_ = 0) into the adjusted sampling level regression model, the predicated sampling level is 4.47%, which is almost exactly the same as the sampling level shown in MATLAB output, 4.5%; moreover, a 95% confidence interval for the predicted sampling level could be able to construct. However, substituting the same data into the unadjusted regression model, the predicated sampling level is 5.25%, far from the optimal solution. From the perspective of sampling level, the adjusted model fits the sampling level very well. Moreover, through the logistic regression analysis, substituting the relevant parameters (*a* = 0.02, *b* = 0.35, *r* = 0.5, *τ*_2_ = 1, and *τ*_4_ = 0) into the estimated model got the probability of sampling level dummy to be 0.029, which is far less than 0.5, indicating that it is worth sampling. This numerical example illustrates that it is necessary to divide the whole experiments into sampling subgroup and nonsampling subgroup and conduct a logistic analysis and a regression analysis if necessary. Moreover, the mean sampling level, in the case of being made samples (0.06013), probably, is more convincing and useful for sales manager than the mean sampling level in the case of all data being included (0.02691).

The above discussions present a two-stage method to improve the accuracy of making sample level involve several operational steps shown below.

**Step 1 .**

Find a similar model based on product-related parameters, and then fit the diffusion equations.

**Step 2 .**

Get the parameters according to the parameter ranges, and compute the simulated data under different situations.

**Step 3 .**

Make statistical analysis for all obtained simulated data, and then take the relevant parameters into logistic regression to check whether to need sampling or not, which is called stage one.

**Step 4 .**

If the result is worth sampling, then take these parameters into adjusted sampling level model to determine the accurate sampling level, which is called stage two.

## 4. Conclusion

Most researches on marketing strategy and on the diffusion of a new product, based on Bass model, generally assumed potential market as a constant or a time function according to product features, lacking the consideration for product diffusion under dynamic potential market. This paper focused on an optimization model for sampling level, comprehensively integrating marketing strategy and consumers' behavior. Global sensitivity analysis, including logistic regression and linear regression analysis, was introduced to discover the impact of variables on output of the diffusion model and to show that a two-stage method is more widely fitted to estimate the relevant parameters in the case of parameters lack of accuracy and to be able to construct a 95% confidence interval for the predicted sampling level.

Considering dynamic potential market, this paper focused on the promotion effect of free samples on the diffusion process based on Bass model. The presented discrete diffusion model is dynamic and flexible so that every situation could be predicted more easily. Then the paper discussed the loss rate of consumers to find the best sampling rate and sampling time. However, the definition of new nondurable product is too ideal to limit the discussion, and there is no reference data to do an empirical analysis. Also, this paper did not consider the impact of pricing strategies and other consumer behaviors, such as multiple unit ownership. Furthermore, the above considerations can also be extended to incorporate multiple-product diffusion process, which might be included in the future work.

## Figures and Tables

**Table 1:** Parameters and parameter level settings.

Parameters	Parameters level
*a*	0.005, 0.01, 0.02, 0.04, 0.08
*b*	0.21, 0.28, 0.35, 0.42, 0.49
*γ*_*u*_	0.01, 0.02, 0.03, 0.04, 0.05
*r*	0.05, 0.10, 0.20, 0.40, 0.80
*τ*	1, 2, 4

**Table 2:** The correlation test with test parameters and decision variables.

Pearson Correlation Coefficients, *N* = 1875 Prob > |*r*| under *H*_0_: *ρ* = 0									
	*a*	*b*	*γ*_*u*_	*r*	*τ*	NPV	sl	*τ*_2_	*τ*_4_
NPV	0.11416	0.10351	−0.05544	0.77334	−0.37394	1	0.64582	−0.04205	−0.31576
	<0.0001	<0.0001	0.0164	<0.0001	<0.0001		<0.0001	0.0687	<0.0001

sl	−0.35051	−0.09772	−0.00821	0.57685	−0.27882	0.64582	1	−0.04268	−0.23167
	<0.0001	<0.0001	0.7224	<0.0001	<0.0001	<0.0001		0.0647	<0.0001

**Table 3:** Statistics of multiple linear regression for sampling level.

Variable	Parameter estimate	*t* value	{*Pr*⁡>|*t*|}
Intercept	0.05213	14.96	<0.0001
*a*	−0.68051	−22.65	<0.0001
*b*	−0.05227	−6.31	<0.0001
*r*	0.11199	37.27	<0.0001
*τ*_2_	−0.02374	−11.83	<0.0001
*τ*_4_	−0.0379	−18.88	<0.0001

Adj. *R*-sq.	0.5511		

**Table 4:** Statistics of multiple linear regression for NPV.

Variable	Parameter estimate	*t* value	{*Pr*⁡>|*t*|}
Intercept	60.7135	3.14	0.0015
sl	2218.66034	20.28	<0.0001
*a*	3152.54911	19.65	<0.0001
*b*	526.37925	13.3	<0.0001
*γ*_*u*_	−1470.49139	−5.37	<0.0001
*r*	864.34291	46.07	<0.0001
*τ*_2_	−169.28447	−17.2	<0.0001
*τ*_4_	−289.812	−27.98	<0.0001

Adj. *R*-sq.	0.8173		

**Table 5:** Testing global null hypothesis.

Test	Chi-square	DF	Pr > ChiSq
Likelihood ratio	1812.8433	6	<0.0001
Score	1032.5284	6	<0.0001
Wald	300.7072	6	<0.0001

**Table 6:** Sampling level dummy by estimation.

sl dummy	Estimation		
	No	Yes	Total
0	949	87	1036
	50.61	4.64	55.25
	91.6	8.4	
	92.5	10.25	

1	77	762	839
	4.11	40.64	44.75
	9.18	90.82	
	7.5	89.75	

Total	1026	849	1875
	54.72	45.28	100

**Table 7:** Analysis of maximum likelihood estimates.

Parameter	DF	Estimate	Standard error	Wald chi-square	Pr > ChiSq
Intercept	1	−1.1695	0.4317	7.3397	0.0067
*a*	1	231.6	14.2897	262.77	<0.0001
*b*	1	−5.2669	0.9571	30.2848	<0.0001
*γ*_*u*_	1	18.2757	6.5105	7.8798	0.005
*r*	1	−13.9136	0.8563	263.9973	<0.0001
*τ*_2_	1	1.4605	0.2398	37.0932	<0.0001
*τ*_4_	1	3.0193	0.2677	127.2064	<0.0001

**Table 8:** Statistics of multiple linear regression for selected sampling experiments.

Variable	Parameter estimate	*t* value	*Pr*⁡>|*t*|
Intercept	0.09514	19.5	<0.0001
*a*	−2.51458	−25.08	<0.0001
*b*	−0.12693	−10.95	<0.0001
*r*	0.18473	41.85	<0.0001
*τ*_2_	−0.04801	−17.87	<0.0001
*τ*_4_	−0.0809	−27.26	<0.0001

Adj. *R*-sq.	0.7472		

## Notation

*t*: Time (from 1 to *T*)
$\bar{N}$: The upper limit of the number of potential consumers
*a*: External influence rate (innovative coefficient)
*b*: Internal influence (imitation coefficient)
*N*(*t*): Cumulative number of the demanders by time *t*
*n*(*t*): The number of the demanders at time *t*
*γ*_*u*_: Change rate of dynamic market
*N*_*f*_(*t*): Cumulative number of the consumers by time *t* who get the free sample but never adopted the product before
*U*(*t*): Cumulative number of loss demanders by time *t*
*u*(*t*): The number of loss demanders at time *t*
*s*(*t*): Sales volume at time *t*
*n*_*f*_(*t*): The number of the consumers who get free samples at time *t*
*τ*: Product life cycle
*r*_*n*_(*t*): Nonadoption ratio of the consumers who get samples
*r*_*b*_: Ratio of potential consumers of new product among the whole consumers
*R*: Repeat purchase rate
*i*_*r*_: Discount rate.
